# Prognostic impact of presumed breast or ovarian cancer among patients with unfavorable-subset cancer of unknown primary site

**DOI:** 10.1186/s12885-018-4092-4

**Published:** 2018-02-13

**Authors:** Makoto Kodaira, Kan Yonemori, Tatsunori Shimoi, Akihiko Yoshida, Masayuki Yoshida, Atsuko Kitano, Akihiko Shimomura, Mayu Yunokawa, Chikako Shimizu, Yuichi Takiguchi, Yasuhiro Fujiwara, Kenji Tamura

**Affiliations:** 1 Department of Breast and Medical Oncology, National Cancer Center Hospital, 5-1-1 Tsukiji, Chuo-ku, Tokyo, 104-0045 Japan; 2 Division of Pathology, National Cancer Center Hospital, 5-1-1 Tsukiji, Chuo-ku, Tokyo, 104-0045 Japan; 3 Department of Medical Oncology, Graduate School of Medicine, Chiba University, 1-8-1 Inohana, Chuo-ku, Chiba, 260–8670 Japan; 4 Department of Medical Oncology, Jikokai Kodaira Hospital, 20-16 Sasame, Minami-cho, Toda, Saitama, 335-0035 Japan

**Keywords:** Cancer of unknown primary site, Unfavorable subset, Presumed primary site

## Abstract

**Background:**

The clinical utility and prognostic impact of presumed primary breast or ovarian cancer among patients with an unfavorable subset of cancer of unknown primary site (CUP) remains unclear. We aimed to evaluate the clinical relevance of the presumed primary site of CUP and the clinical outcome of site-specific therapy based on such presumptions.

**Methods:**

Patients referred to our center who were diagnosed with unfavorable-subset CUP and treated between April 2007 and March 2015 were enrolled in this study. Data were collected retrospectively from the hospital database and electronic medical records. Presumptive primary breast or ovarian cancer was based on histological and immunohistochemical analyses and metastatic patterns. The outcomes of patients with unfavorable-subset CUP with a putative primary site in the breast or ovary (P-CUP) and of patients with unfavorable-subset CUP, but without P-CUP (U-CUP), were assessed.

**Results:**

A total of 780 patients were referred to our hospital with malignancy of unknown origin. Of these, 409 patients were diagnosed with CUP and 344 patients with unfavorable-subset CUP. Following clinicopathological examination, 40 (11.6%) of the 344 patients had P-CUP and the remaining 303 (88.3%) patients had U-CUP. In total, 136 patients received chemotherapy (22 with P-CUP and 114 with U-CUP). Among the 22 patients with P-CUP, three received hormonal therapy for breast cancer, and 19 received chemotherapy based on the presumed primary organ (breast, 4; ovaries, 15). Conventional platinum-based chemotherapy was administered to 105 patients with U-CUP and non-platinum drug treatment to nine patients. The objective response rates were 61.1% (95% confidence interval [CI]: 38.6–83.6) and 41.1% (95% CI: 31.8–50.4) for patients with P-CUP and U-CUP, respectively. The median overall survival durations were 50.0 months and 16.9 months (log-rank: *P* = 0.002) for patients with P-CUP and U-CUP, respectively. P-CUP was identified as an independent predictor of good prognosis according to multivariate analysis.

**Conclusions:**

Patients with P-CUP had higher response rates and a better prognosis compared with patients with U-CUP. It might thus be reasonable to classify this subset as a new category of CUP with a favorable prognosis.

**Electronic supplementary material:**

The online version of this article (10.1186/s12885-018-4092-4) contains supplementary material, which is available to authorized users.

## Background

Cancer of unknown primary site (CUP) is pathologically diagnosed as metastatic carcinoma in which no obvious anatomical primary site is identified after adequate diagnostic evaluation. The prevalence of CUP has been estimated to range from 2%–10% of all malignancies [1, 2]. Etiological data for Japanese patients with CUP is very limited. According to the statistics of The Japanese Ministry of Health, Labour and Welfare, there were an estimated 7000 newly diagnosed cases of malignancy of unknown origin (MUO) covered by ICD-10 code C77-C80 in Japan in 2013, and this number has been gradually increasing [3]. Survival outcomes in CUP patients remain poor, with a median overall survival (OS) of 1.6–13.6 months [4]. Patients with CUP have a poorer prognosis than patients with identified primary sites and those with metastatic cancers with known primary sites [5, 6]. It is therefore essential to identify and/or predict the primary site for all advanced cancer patients who present with metastatic disease in whom the diagnosis of the primary site is uncertain at the time of referral to the oncologic department. Management of these cancers requires a systemic physical examination, focused imaging examinations, and histopathologic analysis [7]. After a comprehensive work-up, patients with CUP with no anatomically defined primary tumor can be divided into two distinct groups: a ‘favorable’ subset and an ‘unfavorable’ subset. Patients in the favorable subset have a favorable response to specific treatments, and are defined by their clinical course, metastatic pattern, and pathologic features [8]. These patients are treated with an approach appropriate for the presumed primary site. The remaining, unfavorable subset of patients have CUP without specific treatment, and have an extremely poor prognosis. Although no standard treatment for this subset has yet been established, drug regimens containing platinum drugs are considered as common empirical treatment for patients with good performance status in daily clinical practice [1]. Additionally, recent improvements in imaging and pathologic diagnostic methods have led to the prediction of the primary site in 20%–25% of CUP patients [7–10], and site-specific therapy for CUP with a putative primary site is thus an attractive strategy. Recent studies have demonstrated that immunohistochemistry (IHC) can identify a unique subset of CUP patients for whom presumed primary site-specific chemotherapy may be beneficial [11, 12]. CUP with a colorectal IHC (CK20+ CDX2+ CK7−) or molecular profile can be reclassified from an unfavorable to a favorable subset [1]. Identification of distinctive subsets of CUP within the unfavorable subset is required to improve the clinical outcomes of patients with CUP. Furthermore, those patients could benefit from the presumptive diagnosis of the primary site by pathological and molecular diagnostic techniques, potentially making them eligible for new and effective therapies for specific cancers. It is necessary to acquire more data on the outcomes of patients with likely primary sites. In a previous study of patients with unknown primary tumors, the identification of specific patient subsets, including patients with identified primary breast or ovarian cancer, contributed to improved survival among patients in whom the primary tumor was found [5]. Among CUP patients with presumed primary breast or ovarian cancer, tumors such as peritoneal adenocarcinomatosis of a serous papillary histological type or isolated axillary nodal metastases in women were categorized in the favorable subset. However, patients with presumed primary breast or ovarian cancer with diffuse metastatic disease remained in the unfavorable subset.

In this study, we aimed to review the diagnostic outcomes of MUOs and the clinical outcomes of patients with unfavorable-subset CUP who received chemotherapy at the National Cancer Center Hospital in Japan. We also assessed the prognostic impact of a presumed primary site in the breast or ovaries in patients with unfavorable-subset CUP.

## Methods

### Patients

Patients who were referred to the National Cancer Center Hospital after being diagnosed with MUO were enrolled in this retrospective study. We evaluated the data for an unfavorable subset of patients with CUP who were treated with.

Data were collected from the National Cancer Center Hospital database and from electronic medical records between April 2007 and March 2015. This study was conducted in accordance with the Declaration of Helsinki and guidelines on Good Clinical Practice and was approved by the local institutional review boards.

### Clinical and pathological work-up of CUP

The initial standard work-up for patients with MUO at the time of their initial visit to the National Cancer Center Hospital Department of Medical Oncology included a detailed medical history, complete physical examination, blood counts, chemistry profile, tumor markers, urine test, chest radiograph, and computed tomography examination from the neck to the pelvis. The following examinations were carried out according to the need for further investigations: endoscopy of suspected areas, urological examination of male patients with elevated prostate-specific antigen levels, breast cancer screening by mammography, ultrasound, and magnetic resonance imaging and gynecological cancer screening by gynecological examination for female patients, and ^18^F–fluorodeoxyglucose positron emission tomography or positron emission tomography/CT. Histopathological review, including immunohistochemistry (IHC) was carried out to identify primary sites in organs. Specific IHC evaluations were carried out when a specific origin was suspected based on morphological examination and clinical history.

### Definition of identified primary site

The diagnosis of a primary tumor required the identification of an appropriate associated physical finding or radiographic or endoscopic findings consistent with a primary tumor at a particular site for common epithelial neoplasms with distinctive pathologic features [5].

### Definition of MUO and CUP

A MUO was defined as a metastatic lesion identified on the basis of a limited number of tests, with no obvious primary site, before comprehensive investigation [2]. A CUP was defined as a metastatic epithelial or neuro-endocrine malignancy identified on the basis of final histology, with no detection of a primary site despite the selected investigations, specialist review, and further specialized investigations as appropriate [2].

### Definition of favorable and unfavorable subsets

The favorable subset of CUP was defined according to the CUP guidelines [1] as a poorly differentiated neuroendocrine carcinoma of an unknown primary site, well-differentiated neuroendocrine carcinoma of an unknown primary site, peritoneal adenocarcinomatosis of a serous papillary histological type in females, isolated axillary nodal metastases in females, squamous cell carcinoma involving nonsupraclavicular cervical lymph nodes, CUP with a colorectal IHC (CK20+ CDX2+ CK7−) or molecular profile, a single metastatic deposit from unknown primary, or blastic bone metastases or IHC/serum prostate-specific antigen expression in male patients. The remaining CUP patients were defined as the unfavorable subset.

### Definition of presumed breast or ovarian primary site

In this study, patients with CUP in the unfavorable subset who had a putative primary breast or ovarian cancer were defined as having CUP with a putative primary site (P-CUP), and the rest were classified as unfavorable subset without specific definition (U-CUP). The presumptive primary site was assessed based on the clinical manifestations, histology, and IHC patterns. In this study, a CUP with putative primary ovarian cancer was defined as adenocarcinoma with positive staining for at least paired-box gene 8 (PAX-8) or Wilm’s tumor protein (WT-1) in female patients. If only PAX-8 was positive, we added thyroid transcription factor-1 (TTF-1) to exclude thyroid cancer. A CUP with positive putative primary breast cancer was defined as adenocarcinoma with positive staining for at least gross cystic disease fluid protein-15 (GCDFP-15) or mammaglobin in female patients. Expression of estrogen receptor (ER), progesterone receptor (PgR), human epidermal growth factor receptor 2 (HER-2) staining, and fluorescence in situ hybridization were assessed for equivalent breast cancer subtypes. We excluded patients in favorable subsets, including women with adenocarcinoma with axillary lymph nodes or with peritoneal carcinomatosis of adenocarcinoma, from the above CUP with presumed primary ovary or breast cancer.

IHC was carried out using the following antibodies: PAX-8 (clone 10,336–1-AP, 1:200; Proteintech, Chicago, IL, USA), WT-1 (clone C-19, 1:500; Santa Cruz Biotechnology, Inc., Paso Robles, CA, USA), TTF-1 (clone 8G7G3/1, 1:100; NeoMarkers, Fremont, CA, USA), GCDFP-15 (clone 23A3, 1:50, Dako, Glostrup, Denmark), mammaglobin (clone 304-1A5,1:200; Dako), ER (clone 1D5, 1:50; Dako), PgR (clone 1A6, 1:50; Dako), and HER-2 (The HercepTest™ kit; Dako).

### Treatment

Treatment in the P-CUP group was based on the oncologic principles established for the management of each primary tumor type. Treatment of patients with presumed breast cancers was based on the intrinsic subtype assessed by IHC according to ER, PgR, and HER-2 expression. Debulking surgery was considered in operable patients with presumed ovarian cancer. Patients with U-CUP were generally treated with platinum-based chemotherapy, depending on the treating physician’s choice.

### Statistical analysis

The primary objective was to evaluate the prognostic impact of presumed primary breast or ovarian cancer as assessed by IHC in CUP patients. Survival curves were estimated using the Kaplan–Meier method and compared using the log-rank test. Prognostic factors were identified by univariate analysis. Cox proportional hazards analysis was then carried out to identify independent prognostic factors. Statistical analyses were carried out using SPSS statistics software (version 22.0; IBM, Chicago, IL, USA).

## Results

### Diagnosis of malignancy of unknown origin

**Fig. 1:**
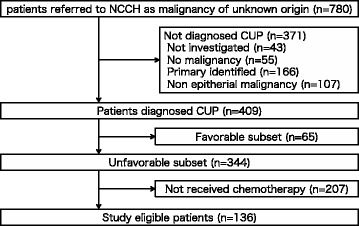
Diagnostic flow chart for patients with malignancy of unknown origin

A total of 780 consecutive patients with suspected MUO were evaluated during the study period. A flow chart showing the diagnostic process is shown in Fig. 1. Forty-three patients (5.6%) were not investigated further, mainly because of advanced age and/or poor performance status. A diagnosis of malignancy could not be established in 55 patients (7.1%). The primary site of the epithelial carcinoma was identified in 166 patients (21.3%) (Additional file 1), and 107 patients (13.7%) were diagnosed with nonepithelial malignancies such as malignant lymphoma, sarcoma, mesothelioma, and others (Additional file 2). Finally, 409 patients were diagnosed with CUP, including 65 (15.9%) patients categorized in the favorable subset (Additional file 3), and 344 patients (84.1%) in the unfavorable subset.

### Patient characteristics of P-CUP and U-CUP

Clinicopathological examination revealed that 40 (11.6%) of the 344 patients had P-CUP and the remaining 303 (87.8%) patients had U-CUP. A total of 136 patients received chemotherapy (P-CUP 22; U-CUP, 114). The median age, sex, performance status, disease distribution, and histological type in the P-CUP and U-CUP groups are listed in Table 1. Seven patients in the P-CUP group, including two men, were presumed to have primary breast cancer and 15 were presumed to have primary ovarian cancer (Additional file 4). Co-expression of GCDFP and mammaglobin were identified in one patient in the P-CUP group with presumed primary breast cancer (Table 2), while the remaining six patients presented with expression of either GCDFP or mammaglobin. The presumed breast cancer subtypes of P-CUP are listed in Table 3. Three patients had hormone receptor-positive and HER-2 negative breast cancer, one patient presented with co-expression of hormone receptors and HER-2, one patient had triple-negative breast cancer, and another patient presented with hormone-negative breast cancer with unknown HER-2 status. Co-expression of WT-1 and PAX-8 was identified in six patients in P-CUP with presumed primary ovarian cancer. The remaining nine patients had tumors expressing either WT-1 or PAX-8 (Table 2).

**Table 1:** Baseline characteristics of patients in the study groups

	P-CUP	U-CUP	
Characteristics	Number		*p*-value
Age, years, median (range)	62 (49–78)	60 (23–79)	
< 65 years	10	34	0.21
> 65 years	12	80	
Sex			
Male	2	60	< 0.001
Female	20	54	
Performance status			
0–1	22	99	0.60
2–4	0	7	
Unknown	0	8	
Site of metastasis			
Visceral organ	4	36	0.31
Lung	2	22	
Liver	2	20	
Brain	0	2	
Other organ	0	7	
Bone	5	25	
Lymph node (LN)			
Superficial LN	14	54	
Deep LN	11	67	
Histology			
Adenocarcinoma	20	56	< 0.001
Non-adenocarcinoma	2	58	
Undifferentiated carcinoma	2	33	
Malignant neoplasm	0	9	
Squamous cell carcinoma	0	16	

Data are presented as n (number), unless otherwise indicated

*P-CUP* CUP with a putative primary site, *U-CUP* patients with unfavorable-subset CUP

**Table 2:** Immunohistochemical profile of P-CUP

	Age	Sex	WT-1	PAX-8	TTF-1	GCDFP-15	Mammaglobin	ER	PgR	HER-2
1	51	F	+	NE	NE	NE	NE	+	NE	NE
2	56	F	+	+	–	–	–	+	NE	NE
3	56	F	+	–	NE	NE	NE	–	–	NE
4	59	F	NE	+	–	NE	NE	NE	NE	NE
5	59	F	–	+	–	NE	NE	–	–	NE
6	61	F	+	NE	–	–	NE	+	–	–
7	61	F	NE	+	–	–	–	+	–	–
8	62	F	–	+	–	–	–	+	NE	NE
9	63	F	+	+	–	–	NE	+	NE	NE
10	66	F	+	+	–	–	–	+	NE	NE
11	69	F	+	+	–	NE	NE	+	–	–
12	69	F	+	+	NE	–	–	NE	NE	NE
13	72	F	+	+	–	–	–	+	+	–
14	73	F	+	NE	NE	–	NE	+	–	–
15	76	F	+	NE	–	–	NE	–	–	–
16	49	F	NE	NE	NE	+	–	+	+	–
17	51	F	–	–	NE	+	–	+	–	–
18	60	F	NE	NE	NE	+	NE	–	–	–
19	66	F	–	NE	–	NE	+	–	–	+
20	68	M	NE	NE	–	+	NE	+	–	+
21	70	M	NE	NE	NE	+	+	–	–	NE
22	72	F	NE	NE	–	+	NE	+	–	–

*WT-1* Wilms’ tumor protein, *PAX-8* paired box gene 8, *TTF-1* thyroid transcription factor-1, *GCDFP-15* gross cystic disease fluid protein-15, *ER* estrogen receptor, *PgR* progesterone receptor, *HER-2* human epidermal growth factor receptor, *NE* not evaluated

**Table 3:** Breast cancer subtype and selected initial treatment for patients with P-CUP with breast cancer features

Breast cancer subtype by using IHC	Number	Treatment given as initial therapy
ER + and or PgR +, and HER-2 -	3	TAM + LH-RH (1), arimidex (2)
ER+ and or PgR +, and HER-2 +	1	PTX + HCN (1)
ER- and PgR –, and HER-2 +	1	CBDCA+PTX + HCN (1)
ER- and PgR-, and HER-2 -	1	CBDCA+PTX
ER- and PgR-, and HER-2 unknown	1	AC (1)

*ER* estrogen receptor, *PgR* progesterone receptor, *HER-2* human epidermal growth factor receptor 2, *TAM* tamoxifen, *LH-RH* luteinizing hormone-releasing hormone analog, *PTX* paclitaxel, *HCN* trastuzumab, *CBDCA* carboplatin, *AC* doxorubicin and cyclophosphamide

### Outcomes of treatment

**Fig. 2:**
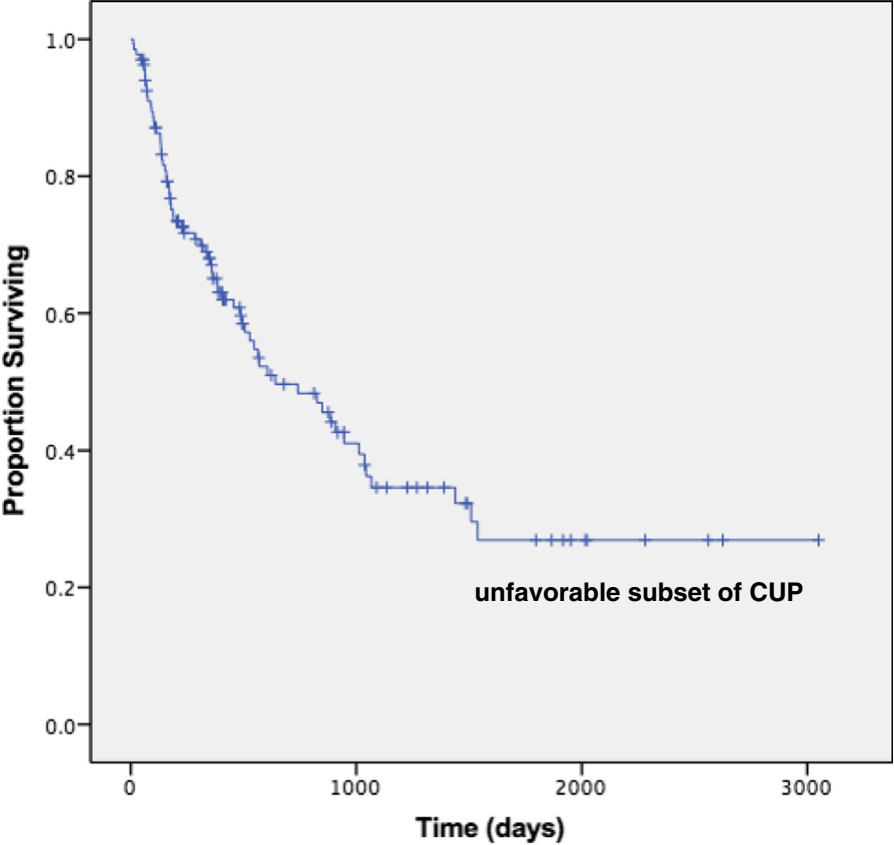
Overall survival of patients with unfavorable-subset CUP treated with chemotherapy

**Fig. 3:**
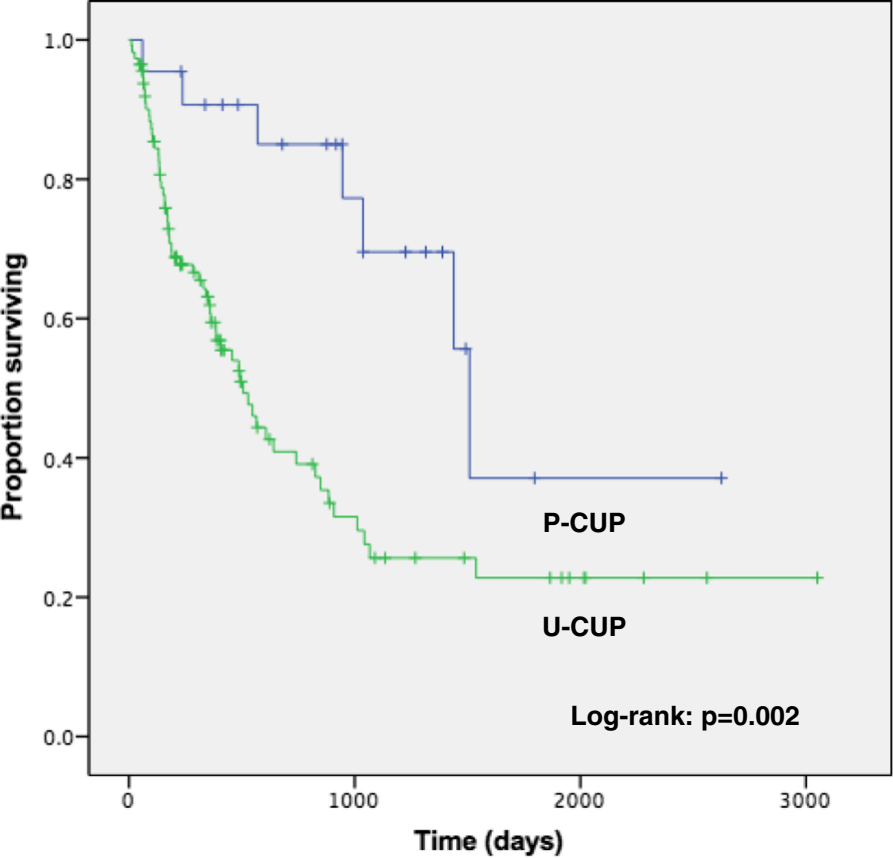
Overall survival of patients in P-CUP and U-CUP groups

Regarding the initial treatment, three patients with P-CUP received hormonal therapy for breast cancer, and the remaining 19 patients received chemotherapy based on the presumed primary site (breast, 4; ovary, 15). Seven of 15 patients with P-CUP with a presumed ovarian primary site underwent debulking surgery. One hundred and five patients with U-CUP were treated with conventional platinum-based chemotherapy, and nine patients received non-platinum drug treatment. The median follow-up time was 12.0 (0.4–100) months. The estimated median OS of all patients with an unfavorable subset of CUP who received chemotherapy was 21.3 months (95% confidence interval [CI]: 11.5–31.2) (Fig. 2). The objective response rates among assessable cases were 61.1% (95% CI: 38.6–83.6) and 41.1% (95% CI: 31.8–50.4) for the P-CUP and CUP groups, respectively. The estimated median OS durations were 50.0 months and 16.9 months for the P-CUP and U-CUP groups, respectively. Kaplan–Meier analysis indicated a significant difference between them (log-rank: *P* = 0.002) (Fig. 3).

### Univariate and multivariate analyses

The clinically relevant covariates (performance status > 2, age > 65 years, sex, adenocarcinoma, visceral metastasis, and P-CUP) were included in a multiple Cox proportional hazards model. P-CUP was identified as an independent risk factor associated with favorable survival (*P* = 0.013), and visceral metastasis was identified as an independent risk factor associated with an unfavorable prognosis (*P* = 0.004) (Table 4).

**Table 4:** Univariate and multivariate analyses

Variable	HR	95% CI	*p*-value
Univariate analysis			
PS > 2	3.30	1.41–7.74	0.006
Age > 65	1.41	0.83–2.39	0.20
Sex (female)	0.76	0.47–1.21	0.25
Visceral metastasis	2.38	1.46–3.88	< 0.001
Adenocarcinoma	1.60	0.98–2.60	0.060
P-CUP	0.32	0.14–0.69	0.004
Multivariate analysis			
PS > 2	1.83	0.75–4.49	0.18
Visceral metastasis	2.21	1.29–3.77	0.004
P-CUP	0.36	0.16–0.81	0.013

*HR* hazard ratio, *CI* confidence interval, *PS* performance status, *P-CUP* CUP with a putative primary site

## Discussion

The ability to identify specific groups among unfavorable subsets of patients with CUP, who might respond favorably to specific therapies is of great interest physicians in terms of considering the optimal treatment strategies. However, the utility of predicting the primary site in patients with unfavorable-subset CUP has not been established. In this study, we evaluated the clinical relevance of the presumed primary site of CUP and the clinical outcomes of site-specific therapies based on the presumed primary site. To the best of our knowledge, this represents the first study to report P-CUP as a favorable prognostic factor in patients with unfavorable subset CUP.

Intention-to-treat analysis demonstrated a median OS of 21.3 months in patients with unfavorable subset CUP, which was better than the previously reported median OS of 9–13 months in patients with good performance status in a phase 2 study evaluating empiric therapies [13]. However, interpretation of published data regarding CUP is challenging, because the lack of a clear and robust definition of CUP has led to apparently wide variances in clinical outcomes. Additionally, according to the definition of CUP, the characteristics of the CUP may vary depending on the diagnostic techniques used. A description of the diagnostic patterns of MUO might thus provide useful information for the interpretation of clinical studies of CUP. In this study, 22 patients with unfavorable-subset CUP were diagnosed with presumed primary breast or ovarian cancer and received chemotherapy. The clinical outcomes of these patients (P-CUP) were excellent, with an estimated median OS of 50.0 months, suggesting a good outcome in this group of unfavorable-subset CUP patients. This study thus provides potentially useful clinical background information regarding the prognosis of CUP with presumed primary breast or ovarian cancer. However, there is limited evidence concerning the treatment selection and clinical outcomes of patients with CUP with presumed primary site, and no data have been reported for patients with unfavorable-subset CUP with presumed breast or ovarian cancer. This might be one reason why P-CUP might be treated as metastatic breast or ovarian cancer, rather than CUP, in daily clinical practice.

IHC stains provide a key diagnostic complement to light microscopy for investigating MUO. In a previous study of CUP, some curable cancers, such as lymphoma and germ cell tumors, which are occasionally confused with CUPs, might have been included in the group of CUPs with poorly differentiated carcinoma [14]. However, the development of pathological diagnostic techniques means that these patients can now be identified and receive appropriate treatment [1]. Recent improvements in diagnostic technologies, including specific IHC stains, allows the identification of patients with highly likely primary sites [8]. However, IHC markers are not uniformly specific or sensitive, and it is therefore important to communicate with the pathologist to ensure the appropriate selection of IHC markers, to avoid using a large series of markers [15]. Several guidelines recommended GCDFP-15 and mammaglobin staining in cases of suspected breast cancer [1, 15], while PAX-8 and WT-1 are recommended IHC markers for CUP with suspected ovarian cancer. However, the above IHC markers are expressed in several cancers and are not completely specific, and it is therefore necessary to be aware of the limitations of the employed IHC markers in clinical use. GCFDFP-15 is expressed in breast cancer, apocrine cancer, and extramammary Paget’s disease [16, 17], and was found to have a sensitivity and specificity for breast cancer of 69.0% and 97.0%, respectively, using the breast cancer clone 23A. Mammaglobin expression is limited in both cancerous and normal breast tissues, and its sensitivity and specificity for breast cancer using 304-1A5 ranged from 50%–70% and 93%–100%, respectively [18]. However, the expression of these markers varies according to histological subtype. The utility of GCDFP-15 and/or mammaglobin is limited in triple-negative breast cancer because of the lack expression of either marker [19]. The expression of GATA-binding protein 3 (GATA-3), a transcription factor involved in the differentiation of breast, urinary organs, skin, and subsets of T lymphocytes, has been reported in urothelial and breast carcinomas, and GATA3 is highly expressed in estrogen receptor-positive breast cancer, as well as estrogen receptor-negative breast cancers, including triple-negative breast cancer [20]. The addition of GATA-3 as a diagnostic marker of CUP might therefore increase the identification of unfavorable-subset P-CUP.

A serous adenocarcinoma histology is a distinctive feature of gynecologic cancers such as ovarian, uterine, and cervical cancer. Nuclear PAX-8 staining is useful for distinguishing between gynecologic cancers and other malignancies, such as malignant mesotheliomas and breast cancer with similar histologic features [21, 22]. Although PAX-8 is also present in renal cancer, thyroid cancers, and pancreatic neuroendocrine tumors [23, 24], TTF-1 is a specific marker for thyroid and lung cancers [25], and a PAX-8 positive, TTF-1 negative adenocarcinoma would exclude thyroid cancer and increase the diagnostic accuracy for gynecologic cancers. Occasional expression of TTF-1 has been reported in ovarian neoplasms, and this should thus be taken into consideration when evaluating adenocarcinomas involving the lung in patients with CUP [26]. We are currently unable to classify the ovarian/renal cancer profile into ovarian and renal cancers by positive PAX-8 expression, because specific markers are not available for renal cancer profiling. However, an adenocarcinoma with morphological features of ovarian or peritoneal serous adenocarcinomas differs from renal cell carcinoma, and we classified ovarian and renal cell carcinomas, other than poorly or undifferentiated adenocarcinomas, as CUP. Serous adenocarcinomas with positive PAX-8 staining can occur at primary sites including the ovary and uterine corpus and cervix [27], making it difficult to identify the anatomical origin in women with genital tract PAX-8-positive serous adenocarcinomas. WT-1 was initially discovered as a tumor suppressor in Wilms’ tumor, and is expressed in most serous adenocarcinomas of the ovary and peritoneum and mesotheliomas, as well as in Wilms’ tumors. WT-1 is therefore useful for confirming the site of origin of serous adenocarcinomas within the female genital tract [28, 29].

We identified a total of 22 patients with P-CUP from the conventional unfavorable subset. In the favorable subset, we identified 19 female patients with peritoneal adenocarcinomatosis of a serous papillary histological type, and ten with isolated axillary nodal metastases. We also identified a total of 51 patients (12.5%) with a presumed primary breast or ovarian cancer in the entire CUP subset. However, it is not clear if P-CUPs are biologically homologous to the favorable subset of CUPs. A previous study at our institution reported on a panel of IHC profiles for the presumed primary site of adenocarcinomas without known origin, using stored tissues samples. In that study, 11 of 71 patients (15.5%) were diagnosed with CUP with breast or ovarian cancer phenotype using the same IHC profile as that used in the current trial [30]. IHC is a simple technique used in daily practice, and the markers described here are useful for making presumptions about the primary site of metastatic adenocarcinoma in CUP. Theoretically, it is difficult to validate the accuracy of IHC for primary organ presumption in CUP, because the primary tumor site is unidentified by definition. Our method was also limited in that it does not allow classification by tissue type (e.g., triple-negative breast cancer, clear cell adenocarcinoma of the ovary, etc.) or specific histological subtype. We can therefore only use IHC to make presumptions about the primary site to allow distinction between patients in the favorable and unfavorable subsets, for which the specific organ features have been identified. However, the presumed primary site does not need to match the true primary site of the CUP itself in clinical practice.

A molecular profiling technique has recently been used for CUP based on validation studies in metastatic cancers with known primaries outside clinical practice [31, 32]. Several commercial assays that classify malignancy of unknown primary origin are currently available for molecular profiling, with reported accuracies of 80%–90% [33–37]. However, molecular profiling is currently not incorporated into clinical practice based on several published clinical guidelines for CUP [1, 15] because of the lack of data regarding their clinical utility. Hainsworth et al. conducted a prospective study of molecular gene expression profiling to predict the tissue of origin and to direct site-specific therapy in patients with CUP, and that patients who received assay-directed site-specific therapy had favorable survival compared with historical controls using empiric CUP regimens [38]. Two prospective clinical trials aimed at evaluating the clinical utility of therapy based on the molecular profiling [NCT00737243, NCT01540058] are currently ongoing. Validation of the accuracy of primary organ predictions using molecular profiling is challenging because, as noted above for IHC, the definition the primary site is unidentified in CUP. However, the presumption of the primary site may be considered reliable if IHC and molecular assays produce identical results.

This study had several limitations. First, the utility of P-CUP as a prognostic factor in CUP was potentially underpowered because the number of patients with each phenotype of breast or ovarian cancer in P-CUP was small, and it was therefore not possible to compare their outcomes with U-CUP. Second, this was a retrospective study and specific IHC staining was only carried out in some patients based on the clinical information and histological subtype at the time of the clinical assessment. However, ad hoc staining of specific markers had a limited impact on the results of this study, because the treatment decision was based on the presumed primary site at the time of diagnosis. Third, the utility of site-specific therapy for P-CUP needs to be studied prospectively by comparing it with standard treatments, such as platinum-containing chemotherapy as the initial therapy. P-CUP with ovarian cancer features would be expected to respond to conventional platinum-containing regimens to some extent, given that the key agents in ovarian cancer treatment are platinum drugs, such as carboplatin. The future development of novel drugs for first-line treatment may mean that the clinical outcomes of patients with P-CUP receiving site-specific therapy might be better than those of patients with P-CUP and U-CUP receiving conventional therapy. Mutation in the breast cancer susceptibility gene (*BRCA*) is a common genetic alteration in ovarian and breast cancers and has been established as a predictive marker for the efficacy of poly (ADP-ribose) polymerase inhibitors [39]. Prospective clinical trials should be conducted to investigate such actionable gene alterations in P-CUP, to optimize chemotherapy and establish the clinical relevance of target genes in P-CUP.

## Conclusions

In conclusion, we identified patients with unfavorable-subset CUP with presumed primary breast or ovarian cancer and treated them with site-specific therapy. This group demonstrated favorable outcomes compared with other patients with unfavorable-subset CUP in whom the presumed primary site could not be identified. Further data are needed to evaluate the survival benefit of the categorization and site-specific therapy based on the presumed primary site in patients with CUP. These results may provide important clinical background data for further clinical investigations of patients with specific unfavorable-subset CUP.

## Additional files

Additional file 1: Primary site identified in patients with MUO. (DOCX 14 kb)

Additional file 2: Nonepithelial malignancy identified in patients with MUO. (DOCX 13 kb)

Additional file 3: Favorable subset identified in CUP according to conventional and new guidelines. (DOCX 14 kb)

Additional file 4: Results of gynecologic examination, mammography, breast ultrasound, breast MRI, and FDG-PET in P-CUP. (DOCX 15 kb)

## Abbreviations

CI: Confidence interval
CUP: Cancer of unknown primary site
ER: Estrogen receptor
GATA3: GATA-binding protein 3
GCDFP-15: Gross cystic disease fluid protein-15
HER-2: Human epidermal growth factor receptor 2
IHC: Immunohistochemistry
MUO: Malignancies of unknown origin
OS: Overall survival
PAX-8: Paired-box gene 8
PgR: Progesterone receptor
TTF-1: Thyroid transcription factor-1
WT-1: Wilms’ tumor protein
