# Supplementary material for: Prognostic impact of presumed breast or ovarian cancer among patients with unfavorable-subset cancer of unknown primary site
Source: BMC Cancer. 2018 Feb 13;18:176. doi: 10.1186/s12885-018-4092-4 (PMC5809895; doi:10.1186/s12885-018-4092-4)
Supplement: Supplementary file 1 — Primary site identified in patients with MUO. (DOCX 14 kb) [file 12885_2018_4092_MOESM1_ESM.docx]

**Additional file 1. Primary site identified in patients with MUO**

| Primary site | Number |
| --- | --- |
| Head and Neck cancer | 5 |
| Thyroid cancer | 3 |
| Lung cancer | 40 |
| Esophageal cancer | 5 |
| Gastric cancer | 10 |
| Colorectal cancer | 16 |
| Hepatobiliary cancer | 8 |
| Pancreatic cancer | 12 |
| Breast cancer | 12 |
| Ovarian cancer | 14 |
| Uterine cancer | 4 |
| Renal cancer | 2 |
| Prostate cancer | 14 |
| Urothelial cancer | 1 |
| Skin cancer | 6 |
| Other type of rare cancer | 14 |

MUO: malignancy of unknown origin
