# Supplementary material for: Prognostic impact of presumed breast or ovarian cancer among patients with unfavorable-subset cancer of unknown primary site
Source: BMC Cancer. 2018 Feb 13;18:176. doi: 10.1186/s12885-018-4092-4 (PMC5809895; doi:10.1186/s12885-018-4092-4)
Supplement: Supplementary file 2 — Nonepithelial malignancy identified in patients with MUO. (DOCX 13 kb) [file 12885_2018_4092_MOESM2_ESM.docx]

**Additional file 2. Nonepithelial malignancy identified in patients with MUO**

| Nonepithelial malignancy | Number |
| --- | --- |
| Malignant lymphoma | 45 |
| Malignant myeloma | 4 |
| Leukemia | 1 |
| Sarcoma | 32 |
| Malignant mesothelioma | 12 |
| Germ cell tumor | 10 |
| Malignant melanoma | 2 |
| Brain tumor | 1 |

MUO: malignancy of unknown origin
