# Supplementary material for: Prognostic impact of presumed breast or ovarian cancer among patients with unfavorable-subset cancer of unknown primary site
Source: BMC Cancer. 2018 Feb 13;18:176. doi: 10.1186/s12885-018-4092-4 (PMC5809895; doi:10.1186/s12885-018-4092-4)
Supplement: Supplementary file 3 — Favorable subset identified in CUP according to conventional and new guidelines. (DOCX 14 kb) [file 12885_2018_4092_MOESM3_ESM.docx]

**Additional file 3. Favorable subset identified in CUP according to conventional and new guidelines**

| Clinical manifestation | Conventional guideline N=55 (13.4%) | New guideline  N=65 (15.9%) |
| --- | --- | --- |
| Poorly differentiated neuroendocrine carcinoma of an unknown primary | 10 | 10 |
| Well differentiated neuroendocrine carcinoma of an unknown primary | 3 | 3 |
| Peritoneal adenocarcinomatosis of a serous papillary histological type in females | 19 | 19 |
| Isolated axillary nodal metastases in females | 10 | 10 |
| Squamous cell carcinoma involving non- supraclavicular cervical lymph nodes | 5 | 5 |
| CUP with a colorectal IHC (CK20+ CDX2+ CK7−) or molecular profile | NE | 10 |
| Single metastatic deposit from unknown primary | 8 | 8 |
| Men with blastic bone metastases or IHC/serum PSA expression | 0 | 0 |

CUP: cancer of unknown primary site; IHC, immunohistochemistry
