# Supplementary material for: Prognostic impact of presumed breast or ovarian cancer among patients with unfavorable-subset cancer of unknown primary site
Source: BMC Cancer. 2018 Feb 13;18:176. doi: 10.1186/s12885-018-4092-4 (PMC5809895; doi:10.1186/s12885-018-4092-4)
Supplement: Supplementary file 4 — Results of gynecologic examination, mammography, breast ultrasound, breast MRI, and FDG-PET in P-CUP. (DOCX 15 kb) [file 12885_2018_4092_MOESM4_ESM.docx]

**Additional file 4. Results of gynecologic examination, mammography, breast ultrasound, breast MRI, and FDG-PET in P-CUP**

|  | Gynecological examination | mammography | Breast ultrasound | Breast MRI | FDG-PET and/or PET/CT |
| --- | --- | --- | --- | --- | --- |
| 1 | (-) | NE | (-) | NE | (-) |
| 2 | (-) | (-) | (-) | NE | (-) |
| 3 | (-) | (-) | (-) | NE | (-) |
| 4 | (-) | NE | (-) | NE | NE |
| 5 | (-) | NE | (-) | NE | (-) |
| 6 | (-) | (-) | (-) | NE | (-) |
| 7 | (-) | NE | NE | NE | (-) |
| 8 | (-) | (-) | (-) | NE | (-) |
| 9 | (-) | (-) | NE | NE | (-) |
| 10 | (-) | (-) | (-) | NE | (-) |
| 11 | (-) | (-) | NE | NE | (-) |
| 12 | (-) | (-) | NE | NE | (-) |
| 13 | (-) | (-) | NE | NE | (-) |
| 14 | (-) | (-) | (-) | (-) | (-) |
| 15 | (-) | (-) | (-) | NE | (-) |
| 16 | (-) | NE | (-) | NE | NE |
| 17 | (-) | NE | (-) | NE | (-) |
| 18 | (-) | (-) | NE | NE | (-) |
| 19 | (-) | (-) | (-) | (-) | (-) |
| 20 | NE | (-) | (-) | NE | (-) |
| 21 | NE | NE | (-) | NE | NE |
| 22 | (-) | (-) | (-) | NE | (-) |

MRI: magnetic resonance imaging, FDG-PET: ¹⁸F-fluorodeoxyglucose positron emission tomography, PET/CT: positron emission tomography/CT, NE: not evaluated, (-): primary site not identified
